# Supplementary material for: TAD border deletion at the Kit locus causes tissue-specific ectopic activation of a neighboring gene
Source: Nat Commun. 2024 May 28;15:4521. doi: 10.1038/s41467-024-48523-7 (PMC11133455; doi:10.1038/s41467-024-48523-7)
Supplement: Supplementary file 1 — Supplementary Information [file 41467_2024_48523_MOESM1_ESM.pdf]

Supplementary notes

Supplementary note 1. Eliminating the contribution of distance decrease to the Hi-C interaction profiles in mutant cells.

In addition to the epigenetic factors, the distribution of genomic contacts is determined by a distance between loci. Since our deletions spanned large genomic regions, we sought to estimate the contribution of distance decrease to the rewiring of chromatin contacts in mutant cells. For this purpose, we generated a custom mm10 genome containing the deletion. Then, we remapped wild-type Hi-C contacts on a custom genome using the C-InterSecture algorithm. The remaining contacts were recalculated according to a P(s) decay and wild-type Hi-C maps with simulated deletions were generated. Then, simulated Wt matrices were used for the estimation of contacts enrichment (Supplementary Fig.7).

Increased inter-TAD interactions are detected on the subtraction maps for Pdgfra Δ2k/Wt (Fig.3c), Pdgfra Δ60k/Wt (Fig.3e), Kit Δ30k/Wt (Fig.4e,f), and KitΔ30k+/Wt (Supplementary Fig.6), indicating that chromatin rewiring was due to the removal of CTCF sites and not to the reduction of genomic distance.

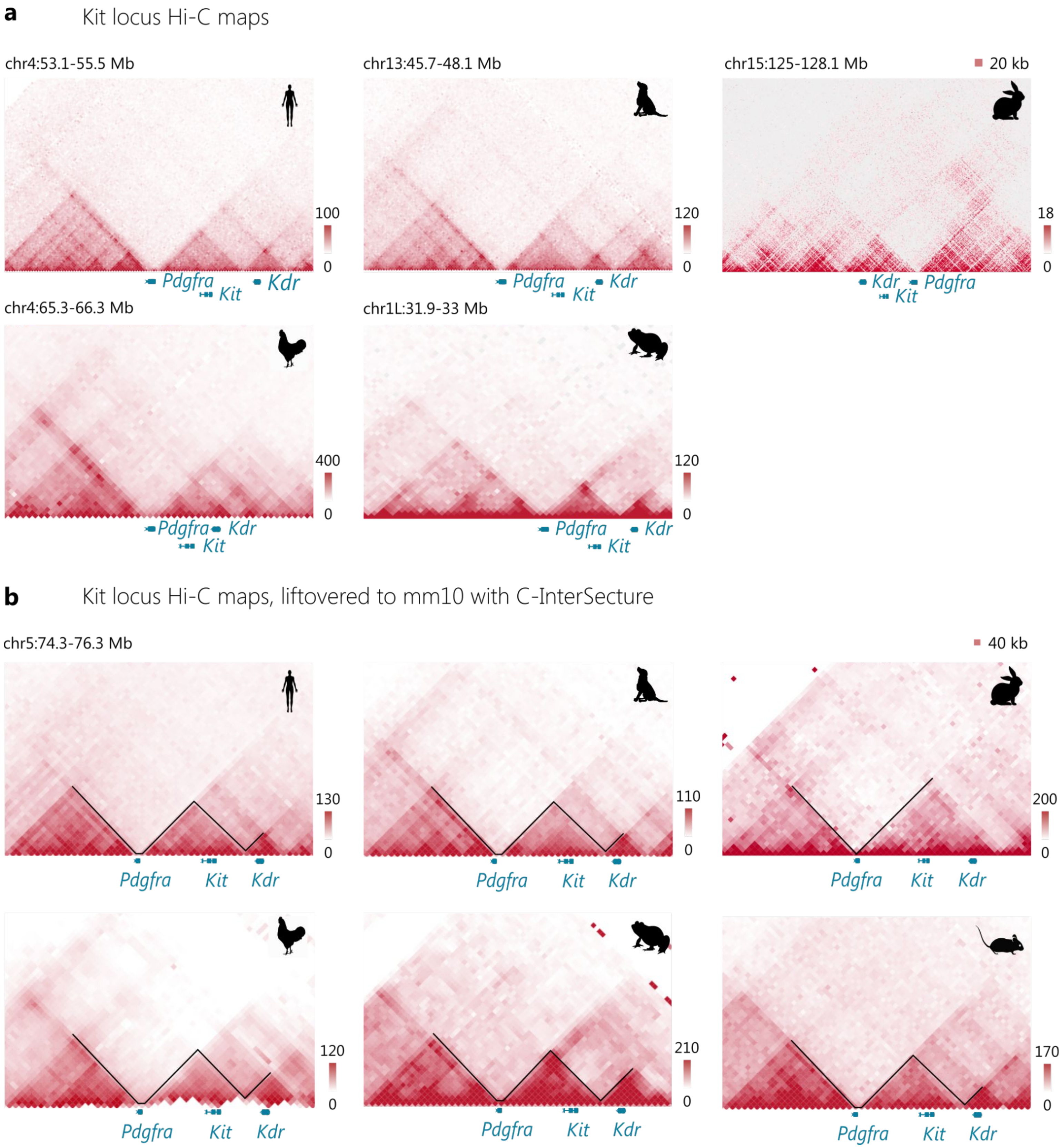

Supplementary Figure 1. Hi-C heatmaps across six vertebrate species demonstrate conservativity of the TADs at the *Kit* locus. (a) Hi-C contacts from human, dog, rabbit, chicken and frog. The Hi-C profile suggests that a synteny break between *Pdgfra* and *Kit* genes, observed in the rabbit data, may be due to a genome assembly error; **(b)** Hi-C contacts liftovered to the mouse genome. The C-InterSecture algorithm was used to neutralize the difference in the genomic distance between syntenic loci in different species. Colour bars reflect the interaction counts.

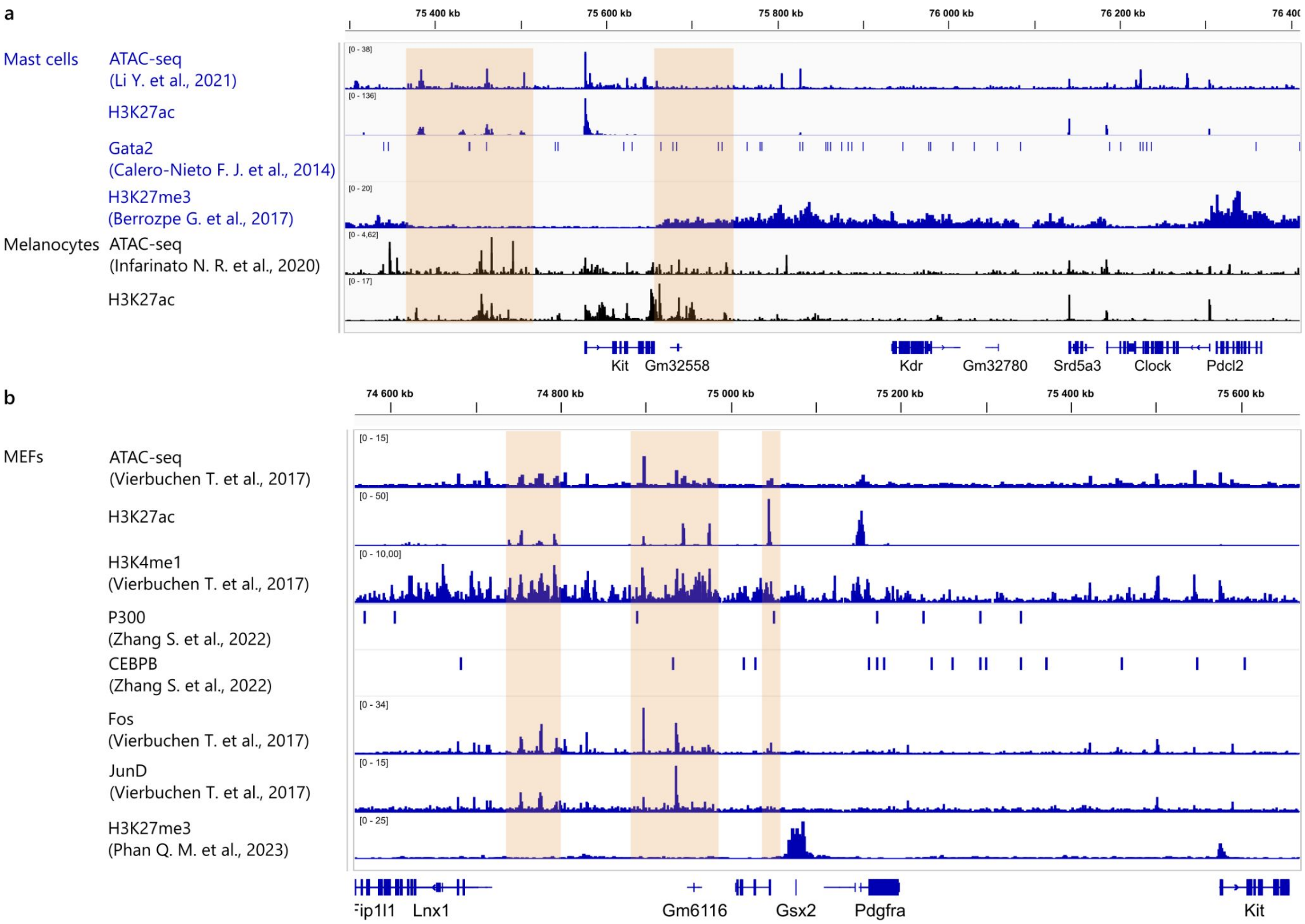

Supplementary Figure 2. Regulatory context of the *Kit* locus in mast cells (a) and MEFs (b). Enrichment of enhancer-associated (H3K27ac, H3K4me1) and heterochromatin-associated (H3K27me3) epigenetic marks, binding of transcription factors (Gata2, Fos, JunD, p300, CEBP), and chromatin accessibility (ATAC-seq). Enhancers highlighted in orange. Apart from H3K27ac data taken from Li Y. et al., 2021, Calero-Nieto F. J. et al., 2014, Berrozpe G. et al., 2017, Infarinato N. R. et al., 2020, Vierbuchen T. et al., 2017, Zhang S. et al., 2022, Phan Q. M. et al., 2023.

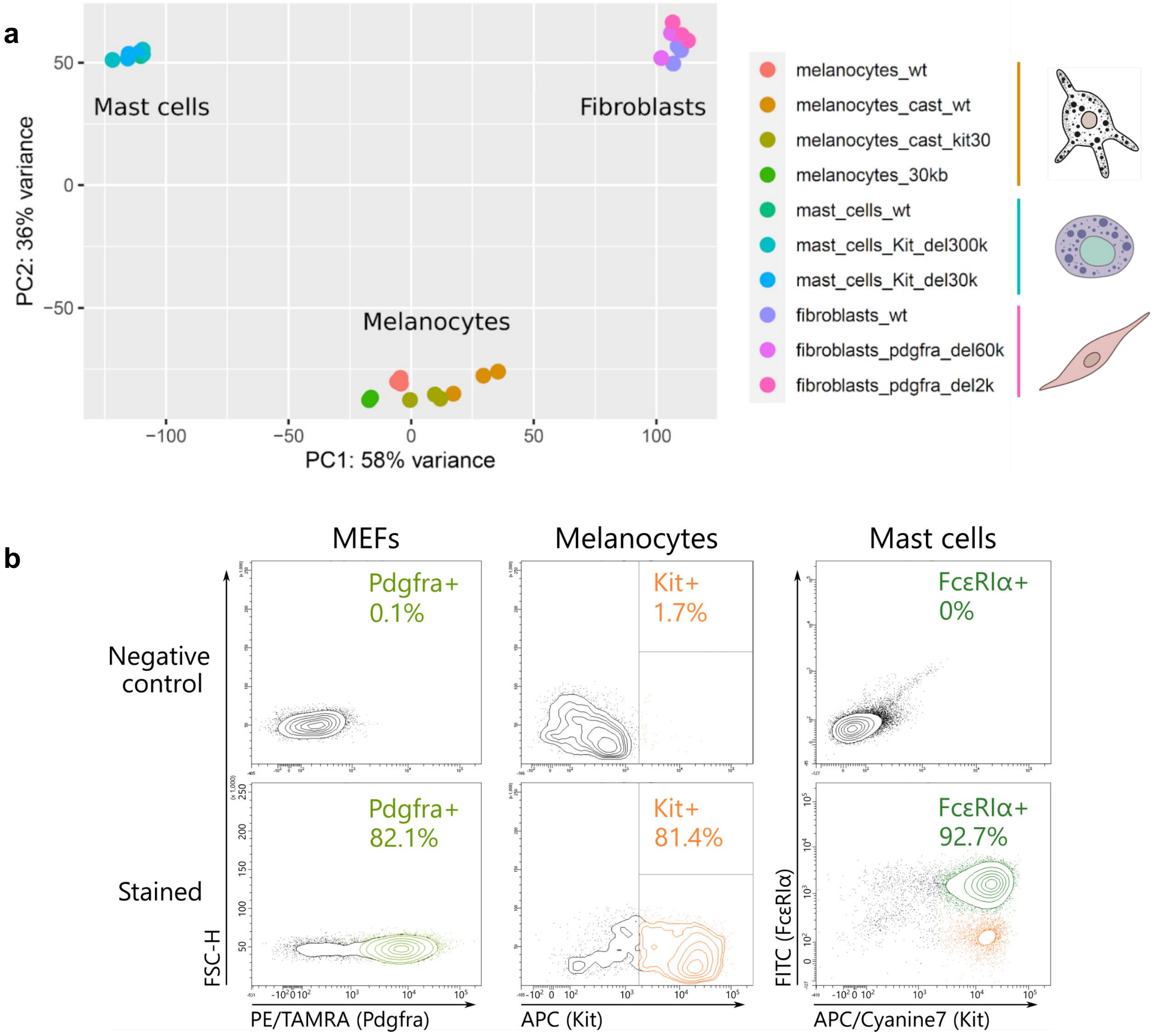

Supplementary Figure 3. RNA-seq and flow cytometry analysis showing the purity of the obtained cell cultures. (a) Plot of the principal component analysis indicates clearly distinguishable transcriptional profiles in the studied cell types and low variability between samples of the same cell type. Colored dots represent individual replicates; **(b)** Flow cytometry plots show average percentage of cell marker-positive cells in a population. Cells were stained with anti-Pdgfra (MEFs), anti-Kit (melanocytes), anti-Kit and anti-FcεRIα (mast cells) antibodies.

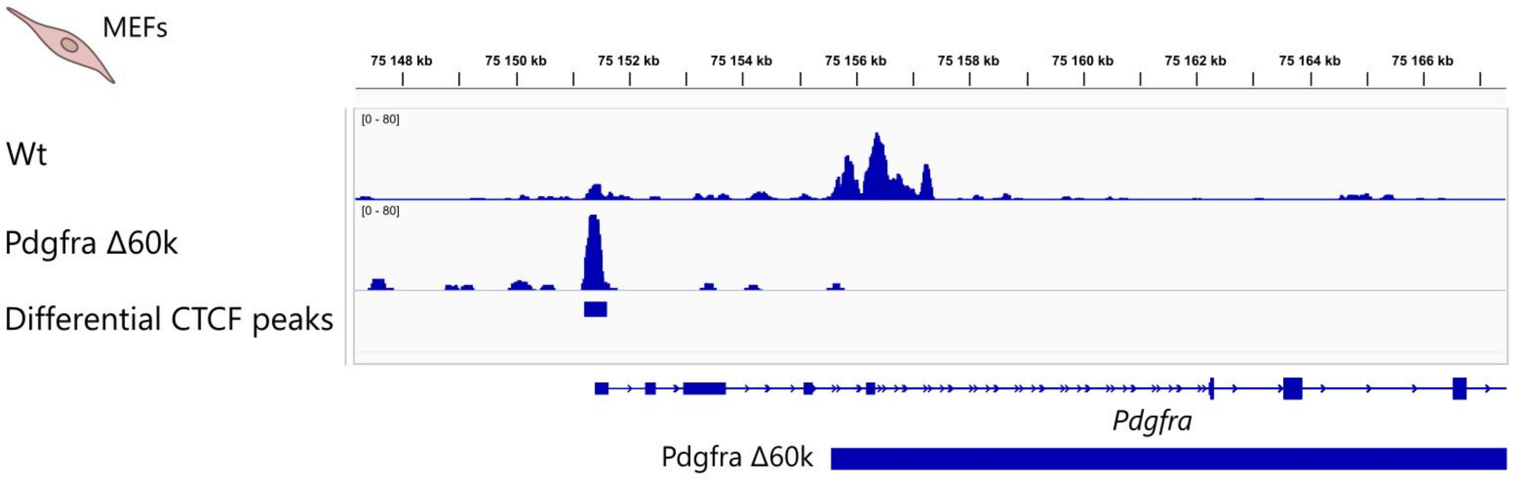

Supplementary Figure 4. Differential CTCF binding peaks analysis at the Pdgfra TAD border. Comparison of CTCF motif at the *Pdgfra* first exon in Wt and Pdgfra Δ60k MEFs reveals increasing CTCF binding in Pdgfra Δ60k, which was confirmed to be differentially enriched by HOMER software (p-value < 0.05).

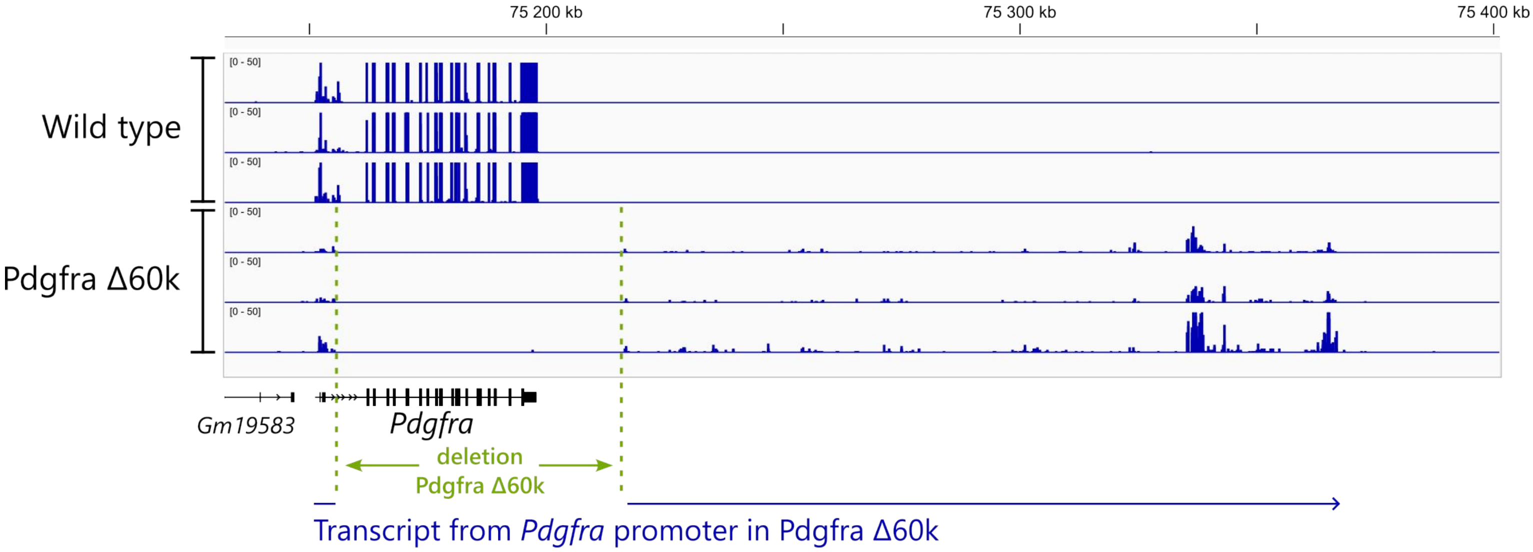

Supplementary Figure 5. Transcription of an intergenic region upon Pdgfra Δ60k deletion in MEFs. Transcriptome analysis using RNA-seq demonstrated an appearance of a new transcript from the *Pdgfra* promoter. The Pdgfra Δ60k did not affect the promoter of the *Pdgfra* gene, but deleted its normal termination site. Thus, a new transcript appeared due to transcription from the promoter continuing to an intergenic region.

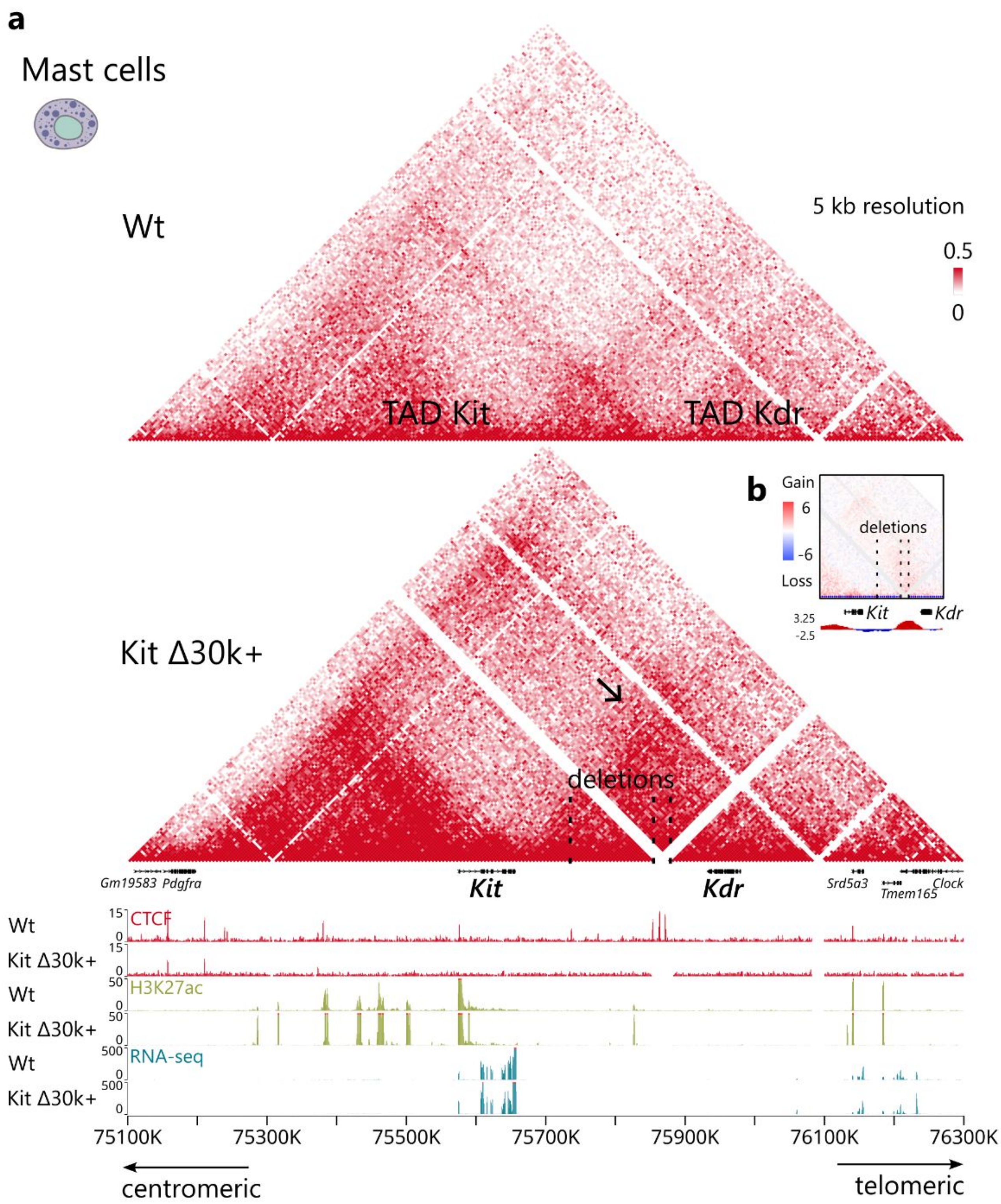

Supplementary Figure 6. A deletion of a CTCF-binding site within the Kit TAD in addition to the Kit/Kdr TADs disruption did not result in *Kdr* activation in mast cells. (a) Removal of boundary resulted in loss of inter-TAD insulation and extensive interactions across the TADs boundary though *Kit* and *Kdr* genes remained insulated, as demonstrated by cHi-C heatmaps, RNA-seq and ChIP-seq signals across the *Kit* locus in wild-type and KitΔ30k+ mast cells. Colour bars reflect the interaction counts. **(b)** A subtraction map (10 kb resolution) of an inter-TADs region demonstrates gain of spatial contacts in KitΔ30k+. The track below the subtraction map shows the estimated contact enrichment, indicating loss of boundary insulation.

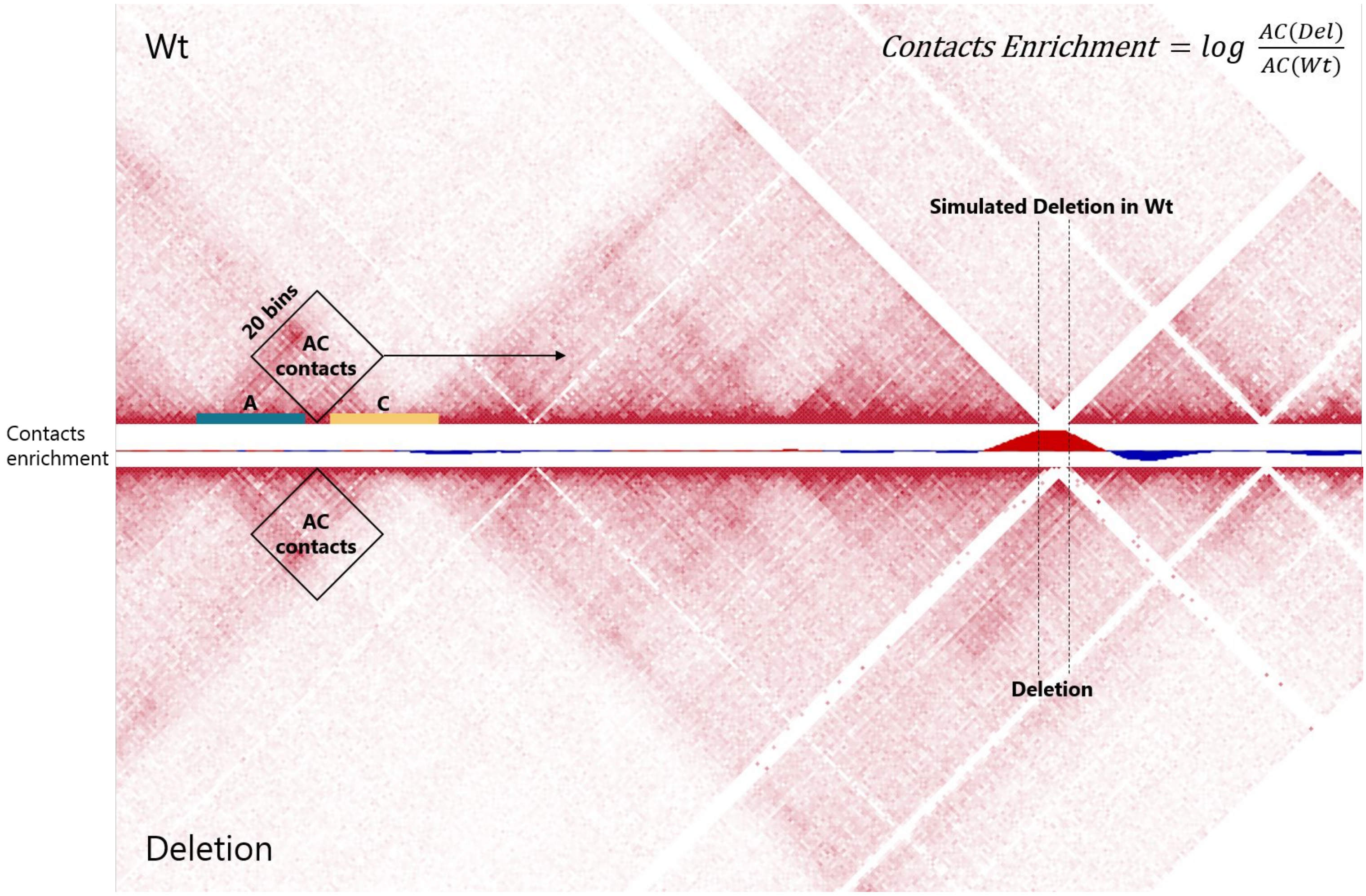

Supplementary Figure 7. Schematic view of the contacts enrichment analysis in the mutant cHi-C data relative to the Wt. Wt cHi-C reads were mapped to a custom genome containing a corresponding deletion. Matrices were VC_SQRT normalized. Then, log2-transformation of each Del contact / Wt contact ratio was performed. Next, we calculated the average contact ratio in a sliding window along the Hi-C diagonal. Obtained values were Z-score normalized. A series of different window sizes were tested (5–40 bins, with 1 bin = 5 kb). A 20 bins window size was used for the analysis.

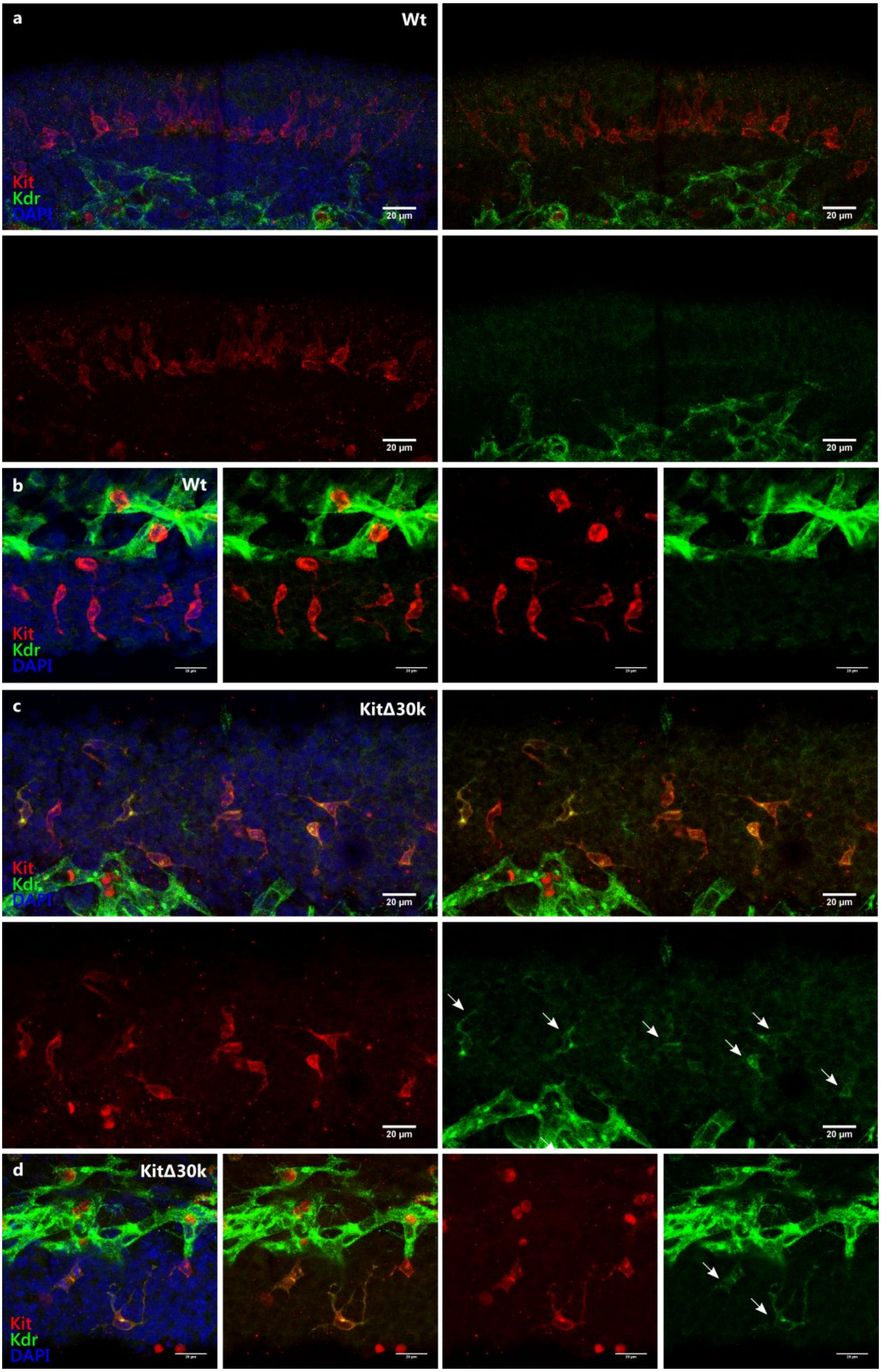

Supplementary Figure 8. Detection of the *Kit-Kdr* double-positive cells in mouse embryonic skin. Skin tissue sections from E15.5 Wt (**a-b**) and Kit Δ30k (**c-d**) embryos. Arrows: double-positive cells (Kit+Kdr+) in Kit Δ30k embryos. Scale bar: 20 um.

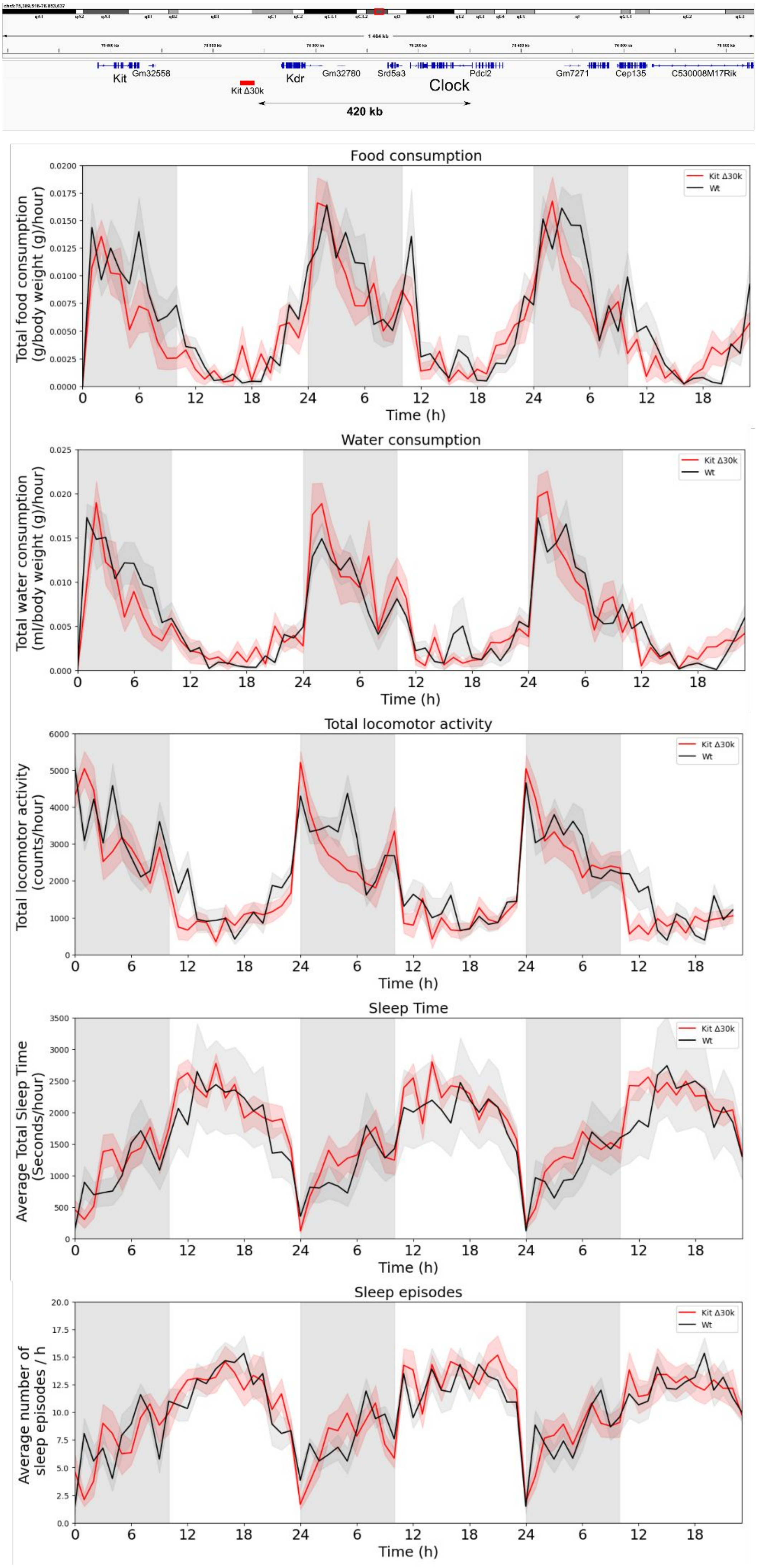

Supplementary Figure 9. Behavioral profile of mice reveals no substantial difference between Wt and KitΔ30k. Total locomotor activity, sleep time and episodes, food and water consumption were measured over the period of three days. The data provided is based on measurements of 12 mice of each genotype.

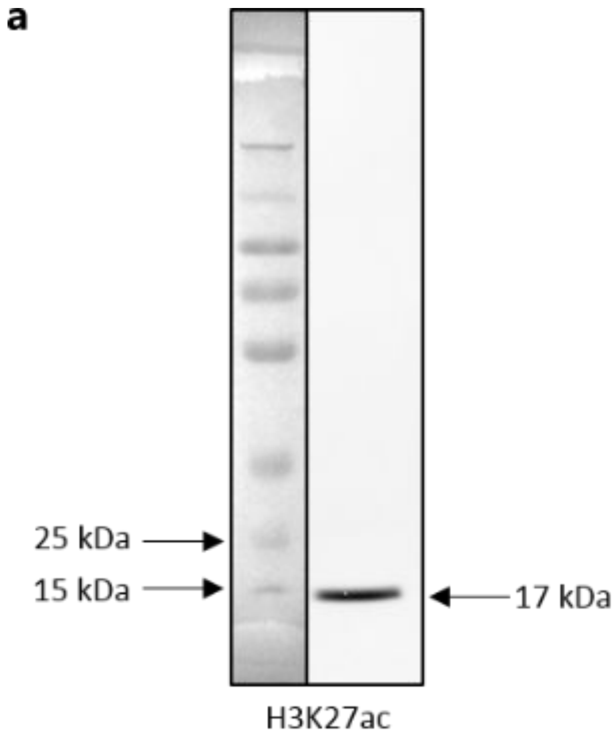

Supplementary Figure 10. Western blot analysis for the antibodies validation. Anti-H3K27ac **(a)** and anti-CTCF **(b)** were diluted 1:3000 for the Western blotting. mESC whole protein samples were used for the western blotting.

Supplementary Table 1. Non-exhaustive list of studies concerning deletions of CTCF binding sites at TADs borders.

Locus	Model	Deletion size	Expression change	Reference
Tbx5/Lhx5, Twist1/Ahr	*In vivo* mouse	21, 13 kb	Yes	Rajderkar S. et al., 2023
Smad7/Smad2, Sim1/Rfx6, Tbx3/Tbx5, Neurog2/Pitx2, Dmrt1,3/Dmrt2	*In vivo* mouse	11–72 kb	No	Rajderkar S. et al., 2023
Sox9/Kcnj2	*In vivo* mouse	6 kb	No*	Despang et al., 2019
HoxD	*In vivo* mouse	26 bp, 1.5 kb	Yes*	Rodríguez-Carballo et al., 2020
HoxD	*In vivo* mouse	~40–90 kb, 350 kb	Yes	Rodríguez-Carballo et al., 2017
HoxD	*In vivo* mouse	~20–30 kb	No	Rodríguez-Carballo et al., 2017
Epha4/Pax3	*In vivo* mouse	1.7 Mb	Yes	Lupiáñez et al., 2015
Shh/Mnx1	mESCs	35 kb	Yes	Williamson et al., 2019
Shh/Mnx1	mESCs	1 kb	No	Williamson et al., 2019
Tsix/Xist	mESCs	58 kb	Yes**	Nora et al., 2012
TAL1/CMPK1; LMO2/CAPRIN1	HEK-293T	400 bp; 25 kb	Yes	Hnisz et al., 2016
HoxA	ESCs, motor neurons	9 bp	Yes	Narendra et al., 2015
PDGFRA	IDH wild-type glioma spheres	10 bp	Yes	Flavahan et al., 2015

* moderate change
** long-range transcriptional misregulation

Supplementary Table 2. List of Guide RNAs and primer sequences for mouse genotyping, used in the study.

gRNA		
**Deletion**	**Protospacer sequence+PAM**	**Mm10 genomic coordinates**
Kit Δ30k	GCGCCGTAAGTGCTGAAAAGAGG<br>GAGGTAAGAGCAATCCGGTAAGG	chr5:75,852,777-75,852,799<br>chr5:75,881,199-75,881,221
Kit Δ30k+	ACAATACAAAATAATCTGGGGGG<br>AAAGCACTATGCCCAGTGAAAGG	chr5:75,736,367-75,736,389<br>chr5:75,736,660-75,736,682
Pdgfra Δ2k	GTGGACCACCAATACTAGCTGGG<br>CAGTGGTTAAGGTCACCGAATGG	chr5:75,155,567-75,155,589<br>chr5:75,157,744-75,157,766
Pdgfra Δ60k	GTGGACCACCAATACTAGCTGGG<br>GTCACCAAGTATGCGGTCATTGG	chr5:75,155,567-75,155,589<br>chr5:75,216,592-75,216,614

Genotyping primers

For Pdgfra/Kit deletions (FWD-278+REV-279; FWD-278+REV-281; FWD-278+REV-308)

Name	Sequence 5'-3'
FWD-278	CTGATTCCCGACCTCATCGG
REV-279	CGTTTGCGACTCGTATCTCG
REV-281	GGACTTTCCTTCCCTCGTCC
REV-308	CTTATGGGCCTCTCGACTCG

For Kit/Kdr deletions (88+89; 90+93)

88	CCTACGAGCCTTCACGTTGT
89	TGAGGACCGCTGATAGGGAA
90	AAGGCTGTTGTACTGCGTGA
93	GTGCTATGGGAGCCGAAAGA

Supplementary references

1. Li, Y. et al. GATA2 regulates mast cell identity and responsiveness to antigenic stimulation by promoting chromatin remodeling at super-enhancers. Nat Commun **12**, 494 (2021).
2. Calero-Nieto, F. J. et al. Key regulators control distinct transcriptional programmes in blood progenitor and mast cells. The EMBO Journal **33**, 1212–1226 (2014).
3. Berrozpe, G. et al. Polycomb Responds to Low Levels of Transcription. Cell Reports **20**, 785–793 (2017).
4. Infarinato, N. R. et al. BMP signaling: at the gate between activated melanocyte stem cells and differentiation. Genes Dev. **34**, 1713–1734 (2020).
5. Vierbuchen, T. et al. AP-1 Transcription Factors and the BAF Complex Mediate Signal-Dependent Enhancer Selection. Molecular Cell **68**, 1067-1082.e12 (2017).
6. Zhang, S. et al. H3K27ac nucleosomes facilitate HMGN localization at regulatory sites to modulate chromatin binding of transcription factors. Commun Biol **5**, 1–14 (2022).
7. Phan, Q. M. et al. Lineage commitment of dermal fibroblast progenitors is controlled by Kdm6b-mediated chromatin demethylation. The EMBO Journal **42**, e113880 (2023).
8. Rajderkar, S. et al. Topologically associating domain boundaries are required for normal genome function. Commun Biol **6**, 1–10 (2023).
9. Despang, A. *et al.* Functional dissection of the Sox9–Kcnj2 locus identifies nonessential and instructive roles of TAD architecture. *Nat Genet* **51**, 1263–1271 (2019).
10. Rodríguez-Carballo, E. *et al.* Chromatin topology and the timing of enhancer function at the HoxD locus. *Proceedings of the National Academy of Sciences* **117**, 31231–31241 (2020).
11. Rodríguez-Carballo, E. et al. The HoxD cluster is a dynamic and resilient TAD boundary controlling the segregation of antagonistic regulatory landscapes. Genes Dev. **31**, 2264–2281 (2017).
12. Lupiáñez, D. G. *et al.* Disruptions of Topological Chromatin Domains Cause Pathogenic Rewiring of Gene-Enhancer Interactions. *Cell* **161**, 1012–1025 (2015).
13. Williamson, I. *et al.* Developmentally regulated Shh expression is robust to TAD perturbations. *Development* **146**, dev179523 (2019).
14. Nora, E. P. *et al.* Spatial partitioning of the regulatory landscape of the X-inactivation centre. *Nature* **485**, 381–385 (2012).
15. Hnisz, D. et al. Activation of proto-oncogenes by disruption of chromosome neighborhoods. Science **351**, 1454–1458 (2016).
16. Narendra, V. *et al.* CTCF establishes discrete functional chromatin domains at the Hox clusters during differentiation. *Science* **347**, 1017–1021 (2015).
17. Flavahan, W. A. *et al.* Insulator dysfunction and oncogene activation in IDH mutant gliomas. *Nature* **529**, 110–114 (2016).
