# Supplementary material for: TAD border deletion at the Kit locus causes tissue-specific ectopic activation of a neighboring gene
Source: Nat Commun. 2024 May 28;15:4521. doi: 10.1038/s41467-024-48523-7 (PMC11133455; doi:10.1038/s41467-024-48523-7)
Supplement: Supplementary file 3 — Reporting Summary [file 41467_2024_48523_MOESM3_ESM.pdf]

nature portfolio

Corresponding author(s): Nariman Battulin

Last updated by author(s): Mar 18, 2024

Reporting Summary

Nature Portfolio wishes to improve the reproducibility of the work that we publish. This form provides structure for consistency and transparency in reporting. For further information on Nature Portfolio policies, see our Editorial Policies and the Editorial Policy Checklist.

Statistics

For all statistical analyses, confirm that the following items are present in the figure legend, table legend, main text, or Methods section.

n/a	Confirmed	
☐	☒	The exact sample size (*n*) for each experimental group/condition, given as a discrete number and unit of measurement
☐	☒	A statement on whether measurements were taken from distinct samples or whether the same sample was measured repeatedly
☐	☒	The statistical test(s) used AND whether they are one- or two-sided<br>*Only common tests should be described solely by name; describe more complex techniques in the Methods section.*
☒	☐	A description of all covariates tested
☐	☒	A description of any assumptions or corrections, such as tests of normality and adjustment for multiple comparisons
☐	☒	A full description of the statistical parameters including central tendency (e.g. means) or other basic estimates (e.g. regression coefficient) AND variation (e.g. standard deviation) or associated estimates of uncertainty (e.g. confidence intervals)
☐	☒	For null hypothesis testing, the test statistic (e.g. *F*, *t*, *r*) with confidence intervals, effect sizes, degrees of freedom and *P* value noted<br>*Give P values as exact values whenever suitable.*
☒	☐	For Bayesian analysis, information on the choice of priors and Markov chain Monte Carlo settings
☒	☐	For hierarchical and complex designs, identification of the appropriate level for tests and full reporting of outcomes
☒	☐	Estimates of effect sizes (e.g. Cohen's *d*, Pearson's *r*), indicating how they were calculated

Our web collection on statistics for biologists contains articles on many of the points above.

Software and code

Policy information about availability of computer code

Data collection

No software was used for data collection.

Data analysis

Software used in the study:
Juicer software Version 1.11 (https://doi.org/10.1016/j.cels.2016.07.002);
C-InterSecture software (https://doi.org/10.1093/bioinformatics/btz415);
LastZ tool Version 1.04.22 (https://github.com/lastz/lastz);
KentUtils (https://github.com/ENCODE-DCC/kentUtils);
Cutadapt tool (https://doi.org/10.14806/ej.17.1.200);
FastQC Galaxy Version 0.73 (https://www.bioinformatics.babraham.ac.uk/projects/fastqc/);
AQUAS ChIP-Seq pipeline1 (https://github.com/kundajelab/chipseq_pipeline);
FIMO tool Version 5.5.0 (https://doi.org/10.1093/nar/gkv416);
RNA STAR Galaxy Version 2.7.8a (https://doi.org/10.1093/bioinformatics/bts635);
featureCounts Galaxy Version 2.0.1 (https://doi.org/10.1093/bioinformatics/btt656);
DESeq2 Galaxy Version 2.11.40.7 (https://doi.org/10.1186/s13059-014-0550-8);
HOMER software Version 4.11 (https://doi.org/10.1016/j.molcel.2010.05.004).

For manuscripts utilizing custom algorithms or software that are central to the research but not yet described in published literature, software must be made available to editors and reviewers. We strongly encourage code deposition in a community repository (e.g. GitHub). See the Nature Portfolio guidelines for submitting code & software for further information.

Data

Policy information about availability of data

All manuscripts must include a data availability statement. This statement should provide the following information, where applicable:

- Accession codes, unique identifiers, or web links for publicly available datasets
- A description of any restrictions on data availability
- For clinical datasets or third party data, please ensure that the statement adheres to our policy

The raw sequencing data have been deposited in the NCBI SRA database with the following accession number PRJNA838252. Processed data, including Hi-C contact maps, RNA-seq and ChIP-seq tracks are available at https://genedev.bionet.nsc.ru/ftp/by_Project/Kit_locus_GEO.

The data used for other organisms are publicly available and can be found under the following web links:
Fibroblasts of human, mouse, rabbit, dog and chicken (https://doi.org/10.1186/s12915-022-01301-7);
African clawed frog fibroblasts (https://doi.org/10.1126/science.abe2218).

Research involving human participants, their data, or biological material

Policy information about studies with human participants or human data. See also policy information about sex, gender (identity/presentation), and sexual orientation and race, ethnicity and racism.

Reporting on sex and gender	N/A
Reporting on race, ethnicity, or other socially relevant groupings	N/A
Population characteristics	N/A
Recruitment	N/A
Ethics oversight	N/A

Note that full information on the approval of the study protocol must also be provided in the manuscript.

Field-specific reporting

Please select the one below that is the best fit for your research. If you are not sure, read the appropriate sections before making your selection.

☒ Life sciences ☐ Behavioural & social sciences ☐ Ecological, evolutionary & environmental sciences

For a reference copy of the document with all sections, see nature.com/documents/nr-reporting-summary-flat.pdf

Life sciences study design

All studies must disclose on these points even when the disclosure is negative.

Sample size	The main factor determing samples size was a sufficient DNA quantity for experiment manipulations and NGS-sequencing. We determined the number of replicates based on common standards in genomic studies and ENCODE guidelines (https://www.encodeproject.org/about/experiment-guidelines/), such as at least 2 biological replicates for ChIP-seq, RNA-seq, and Hi-C. Accordingly, sample size was at least 2 homozygous mice to obtain primary mast cells, 2 homozygous mouse embryos to obtain primary fibroblasts, 8 mouse pups to obtain 2 replicates of primary melanocytes. We did not apply statistical methods to pre-determine the sample size and followed the general standard practice in the field.
Data exclusions	No data were excluded from the analysis.
Replication	All Hi-C experiments were repeated at least in 2 replicates, ChIP-seq experiments repeated at least in 2 replicates, RNA-seq experiments repeated at least in 3 replicates. All attempts at replication were successful and gave reproducible experimental findings. Flow cytometry and immunohistochemistry results were confirmed by several (at least 2) independent attempts.
Randomization	Randomization was not relevant for the study because all the experiments performed had to take into account the genotype of the samples and the study did not involve treatment nor other covariates were expected to introduce biases.
Blinding	The blinding was not relevant for our study because mouse breeding requires knowledge of their genotypes and other analyses are quantitative and rely on data-driven procedures, which did not require subjective interpretation.

Reporting for specific materials, systems and methods

We require information from authors about some types of materials, experimental systems and methods used in many studies. Here, indicate whether each material, system or method listed is relevant to your study. If you are not sure if a list item applies to your research, read the appropriate section before selecting a response.

Materials & experimental systems

n/a	Involved in the study	
☐	☒	Antibodies
☐	☒	Eukaryotic cell lines
☒	☐	Palaeontology and archaeology
☐	☒	Animals and other organisms
☒	☐	Clinical data
☒	☐	Dual use research of concern
☒	☐	Plants

Methods

n/a	Involved in the study	
☐	☒	ChIP-seq
☐	☒	Flow cytometry
☒	☐	MRI-based neuroimaging

Antibodies

Antibodies used	ChIP-seq:<br>Anti-Histone H3 (acetyl K27), Abcam, #ab4729 (polyclonal); 1.5-5 µg for ChIP, 1:3000 for Western Blot<br>Anti-CTCF, Abcam, #ab70303 (polyclonal); 1.5-5 µg for ChIP, 1:3000 for Western Blot<br><br>Immunohistochemistry (IHC):<br>Mouse VEGFR2/KDR/Flk-1, R&D Systems, #AF644 (polyclonal), dilution 1:100<br>c-Kit (D13A2) XP® Rabbit mAb, CellSignaling, #3074 (monoclonal, clone: D13A2), dilution 1:100<br>Cy™3 AffiniPure Donkey Anti-Goat, Jackson ImmunoResearch, #705-165-147, dilution 1:500<br>Alexa Fluor® 488 AffiniPure Donkey Anti-Rabbit, Jackson ImmunoResearch, #711-545-152, dilution 1:500<br><br>Flow cytometry:<br>APC/Cyanine7 anti-mouse CD117 (c-Kit), BioLegend, #135136 (monoclonal, clone: ACK2), dilution 1:300<br>APC anti-mouse CD117 (c-Kit), BioLegend, #105812 (monoclonal, clone: 2B8), dilution 1:300<br>PE anti-mouse CD140a (Pdgfra), BioLegend, #135905 (monoclonal, clone: APA5), dilution 1:300<br>FITC anti-mouse FcεRIα, BioLegend, #134306 (monoclonal, clone: MAR-1), dilution 1:300
Validation	We have successfully validated antibodies for ChIP-seq by Western Blot analysis. WB image is provided in Supplementary Materials (Supplementary Fig.10).<br>Antibodies for IHC and FACS have been validated by the manufactures for the species (mouse) and applications (IHC or FACS) by the correspondent manufacturer. See specifity statement from the manufactures websites accordingly:<br>Mouse VEGFR2/KDR/Flk-1 (https://www.rndsystems.com/products/mouse-vegfr2-kdr-flk-1-antibody_af644),<br>c-Kit (D13A2) XP® Rabbit mAb (https://www.cellsignal.com/products/primary-antibodies/c-kit-d13a2-xp-rabbit-mab/3074),<br>APC/Cyanine7 anti-mouse CD117 (c-Kit) (https://www.biolegend.com/nl-nl/products/apc-cyanine7-anti-mouse-cd117-c-kit-antibody-13795),<br>APC anti-mouse CD117 (c-Kit) (https://www.biolegend.com/en-gb/products/apc-anti-mouse-cd117-c-kit-antibody-72?GroupID=BLG1945),<br>PE anti-mouse CD140a (Pdgfra) (https://www.biolegend.com/de-de/products/pe-anti-mouse-cd140a-antibody-6253?GroupID=BLG8105),<br>FITC anti-mouse FcεRIα (https://www.biolegend.com/fr-ch/products/fitc-anti-mouse-fcepsilonrialpha-antibody-5949?GroupID=BLG6716).

Eukaryotic cell lines

Policy information about cell lines and Sex and Gender in Research

Cell line source(s)	All primary cell lines were obtained for the study from mouse embryos (fibroblasts), skin tissues (melanocytes), and bone marrow (mast cells). Sex of embryos was not determined, melanocytes and mast cells cultures were obtained from male mice.
Authentication	Mast cell cultures were authenticated by toluidine blue staining. All cell cultures obtained had distinct morphology, such as granules of dark pigment in melanocytes. Flow cytometry was used to confirm an appropriate expression of membrane receptors.
Mycoplasma contamination	The cell lines were not tested for mycoplasma contamination.
Commonly misidentified lines<br>(See ICLAC register)	No commonly misidentified cell lines were used.

Animals and other research organisms

Policy information about studies involving animals; ARRIVE guidelines recommended for reporting animal research, and Sex and Gender in Research

Laboratory animals	Animals were kept in a standard environment at 24°C temperature, 40–50% relative air humidity and 14 h light/10 h dark–light-cycle. Food and water were available ad libitum. At the end of experiments, remaining animals were euthanized by CO2.<br><br>Mouse lines described in the study were established in a C57BL/6J background (Mus musculus): Pdgfra Δ2k, Pdgfra Δ60k, Kit Δ30k, Kit Δ30k+, Kit Δ300k. Kit Δ30k line was established from a chimeric animal generated after injection of mouse embryonic stem cells, which were derived from 129S2/SvPasCrl mouse strain. Mice of various ages were used for the study: E13.5 embryos, 4-day-old pups, adult mice.<br><br>Mouse strain CAST/EiJ (Mus castaneus) was used to breed with Wild type and Kit Δ30k strain. 4-day-old hybrid pups were used for the study.<br><br>All the procedures and technical manipulations with animals were in compliance with the European Communities Council Directive of 24 November 1986 (86/609/EEC) and approved by the Bioethical Committee at the Institute of Cytology and Genetics (Permission N45 from 16 November 2018).
Wild animals	The study did not involve wild mice.
Reporting on sex	Sex was not considered in the study design because the study concerns the chromatin structure of an autosome and did not involve analysis of sex-associated genes.
Field-collected samples	The study did not involve samples collected from the field.
Ethics oversight	Bioethical Committee at the Institute of Cytology and Genetics SB RAS

Note that full information on the approval of the study protocol must also be provided in the manuscript.

Plants

Seed stocks	N/A
Novel plant genotypes	N/A
Authentication	N/A

ChIP-seq

Data deposition

☒ Confirm that both raw and final processed data have been deposited in a public database such as GEO.

☒ Confirm that you have deposited or provided access to graph files (e.g. BED files) for the called peaks.

Data access links<br>*May remain private before publication.*	ChIP-seq raw reads: https://www.ncbi.nlm.nih.gov/sra/?term=PRJNA838252+chip<br>ChIP-seq processed reads are available at https://genedev.bionet.nsc.ru/ftp/by_Project/Kit_locus_GEO/ChIP-seq/
Files in database submission	Mast cells del30k H3K27ac<br>210616_I100400190019_V350019587_L3_VL5_1.fq.gz<br>210616_I100400190019_V350019587_L3_VL5_2.fq.gz<br>Mast cells del30k H3K27ac input<br>210616_I100400190019_V350019587_L3_VL6_1.fq.gz<br>210616_I100400190019_V350019587_L3_VL6_2.fq.gz<br>Mast cells del30k+ H3K27ac<br>kit30+_h3k27ac_2_L1_1.fq.gz<br>kit30+_h3k27ac_2_L1_2.fq.gz<br>Mast cells del30k+ H3K27ac input<br>input_h3k27ac_2_L1_1.fq.gz<br>input_h3k27ac_2_L1_2.fq.gz<br>Mast cells WT H3K27ac<br>210616_I100400190019_V350019587_L4_VL3_1.fq.gz<br>210616_I100400190019_V350019587_L4_VL3_2.fq.gz

Mast cells WT H3K27ac input
210616_I100400190019_V350019587_L4_VL4_1.fq.gz
210616_I100400190019_V350019587_L4_VL4_2.fq.gz
MEF del2k H3K27ac
210616_I100400190019_V350019587_L4_EK2_1.fq.gz
210616_I100400190019_V350019587_L4_EK2_2.fq.gz
MEF del2k input
210616_I100400190019_V350019587_L4_EK3_1.fq.gz
210616_I100400190019_V350019587_L4_EK3_2.fq.gz
MEF del60k H3K27ac
210616_I100400190019_V350019587_L4_EK5_1.fq.gz
210616_I100400190019_V350019587_L4_EK5_2.fq.gz
MEF del60k H3K27ac input
210616_I100400190019_V350019587_L4_EK6_1.fq.gz
210616_I100400190019_V350019587_L4_EK6_2.fq.gz
MEF del2k CTCF
210616_I100400190019_V350019587_L4_EK1_1.fq.gz
210616_I100400190019_V350019587_L4_EK1_2.fq.gz
Mast cells WT CTCF
wt CTCF_R1.fastq.gz
wt CTCF_R2.fastq.gz
Mast cells WT CTCF input
wt input_R1.fastq.gz
wt input_R2.fastq.gz
Mast cells del30k CTCF
A_chip30_single_1_NR7.fq.gz
A_chip30_single_2_NR7.fq
Mast cells del30k CTCF input
A_input30_single_1_NR6.fq.gz
A_input30_single_2_NR6.fq.gz
Mast cells del30k+ CTCF
A_chip30p_single_1_NR15.fq.gz
A_chip30p_single_2_NR15.fq.gz
Mast cells del30k+ CTCF input
A_input30p_single_1_NR13.fq.gz
A_input30p_single_2_NR13.fq.gz
Mast cells del300k H3K27ac
210616_I100400190019_V350019587_L4_VL1_1.fq.gz
210616_I100400190019_V350019587_L4_VL1_2.fq.gz
Mast cells del300k H3K27ac input
210616_I100400190019_V350019587_L4_VL2_1.fq.gz
210616_I100400190019_V350019587_L4_VL2_2.fq.gz
Melanocytes Wt H3K27ac
mel_chip_L1_1.fq.gz
mel_chip_L1_2.fq.gz
Melanocytes Wt H3K27ac input
mel_input_L1_1.fq.gz
mel_input_L1_2.fq.gz
Melanocytes del30k H3K27ac
c_L1_1.fq.gz
c_L1_2.fq.gz
Melanocytes del30k H3K27ac input
f_input_L1_1.fq.gz
f_input_L1_2.fq.gz
MEF del60k CTCF (rep1)
rep_1_P_KO_CTCF_R1.fastq.gz
rep_1_P_KO_CTCF_R2.fastq.gz
MEF del60k CTCF input (rep1)
rep_1_P_KO_input_R1.fastq.gz
rep_1_P_KO_input_R2.fastq.gz
MEF del60k CTCF (rep2)
rep_2_P_KO_CTCF_R1.fastq.gz
rep_2_P_KO_CTCF_R2.fastq.gz
MEF del60k CTCF input (rep2)
rep_2_P_KO_input_R1.fastq.gz
rep_2_P_KO_input_R2.fastq.gz
MEF wt CTCF (rep1)
sample-2-2_R1_001.fastq.gz
sample-2-2_R2_001.fastq.gz
MEF wt CTCF input (rep1)
sample-2-7_R1_001.fastq.gz
sample-2-7_R2_001.fastq.gz
MEF wt CTCF (rep2)
sample-2-5_R1_001.fastq.gz
sample-2-5_R2_001.fastq.gz
MEF wt CTCF input (rep2)

sample-2-14_R1_001.fastq.gz
sample-2-14_R2_001.fastq.gz
MEF wt H3K27ac (rep1)
sample-2-10_R1_001.fastq.gz
sample-2-10_R2_001.fastq.gz
MEF wt H3K27acinput (rep1)
sample-2-12_R1_001.fastq.gz
sample-2-12_R2_001.fastq.gz
MEF wt H3K27ac (rep2)
sample-2-6_R1_001.fastq.gz
sample-2-6_R2_001.fastq.gz
MEF wt H3K27ac input (rep2)
sample-2-16_R1_001.fastq.gz
sample-2-16_R2_001.fastq.gz

Genome browser session (e.g. UCSC): IGV, Juicebox, WashU Epigenome Browser

Methodology

Replicates: 2

Sequencing depth: Paired-end reads, 150 bp

Antibodies: Anti-Histone H3 (acetyl K27), Abcam, #ab4729;
Anti-CTCF, Abcam, #ab70303.

Peak calling parameters: AQUAS pipeline (https://github.com/kundajelab/chipseq_pipeline) with the SPP (for CTCF binding analysis) or MACS2 (for histone modifications), default parameters.

Data quality: To assess the ChIP-seq enrichment we used metrics, provided by the SPP (for CTCF binding analysis) or MACS2 (for histone modifications) peak callers. Library complexity, Irreproducible Discovery Rate and Fingerprint plots of ChIP vs. Input was used to ensure quality of the libraries.

Software: FastQC Galaxy (Version 0.73);
AQUAS ChIP-Seq pipeline1 (https://github.com/kundajelab/chipseq_pipeline);
FIMO tool (Version 5.5.0).

Flow Cytometry

Plots

Confirm that:

☒ The axis labels state the marker and fluorochrome used (e.g. CD4-FITC).

☒ The axis scales are clearly visible. Include numbers along axes only for bottom left plot of group (a 'group' is an analysis of identical markers).

☒ All plots are contour plots with outliers or pseudocolor plots.

☒ A numerical value for number of cells or percentage (with statistics) is provided.

Methodology

Sample preparation: A total of ~0.5-1 mln cells/ml were harvested. Antibody and cell suspension were mixed in the buffer (PBS, 10% FBS) in a ratio 1:100 and incubated on ice for 1 hour. Before FC cell suspension was filtered through nylon mesh to remove cell conglomerates. A negative control was prepared as a cell suspension washed in PBS without immunostaining.

Instrument: BD FACSAria III (BD Biosciences)

Software: BD FACSDiva™ Software (BD Biosciences)

Cell population abundance: No sorting was performed for this study.

Gating strategy: First, we used forward and side scatter density plots for identifying cell populations of interest and excluding debris. Second, cells were gated against forward scatter height versus forward scatter area density plot for doublet exclusion from the dataset. Third, we used single parameter histograms for identifying cells with/without specific marker expression and two parameter density plots for detailed analysis.

☐ Tick this box to confirm that a figure exemplifying the gating strategy is provided in the Supplementary Information.
